# Tracking the surface structure and the influence of cations and anions on the double-layer region of a Au(111) electrode†

**DOI:** 10.1039/d4cp02133a

**Published:** 2024-08-01

**Authors:** Ariba Adnan, Saeid Behjati, Núria Félez-Guerrero, Kasinath Ojha, Marc T. M. Koper

**Affiliations:** a Leiden Institute of Chemistry, Leiden University Einsteinweg 55 Leiden 2333 CC The Netherlands m.koper@chem.leidenuniv.nl; b Chemistry Department, Universitat Autònoma de Barcelona Bellaterra-08193 Barcelona Spain

## Abstract

We examined the electric double-layer (EDL) of a Au(111) electrode in a dilute perchloric acid solution using a combination of capacitance measurements and *in situ* scanning tunnelling microscopy under electrochemical conditions (ECSTM). The “camel-shaped” capacitance curve of the EDL is studied with different cations and anions, including their impact on the potential of zero charge (PZC). We show that the ECSTM images of thermally reconstructed and of the potential-induced surface reconstruction of Au(111) in perchloric acid electrolyte resemble previous work in sulphuric acid, displaying a herringbone pattern for a thermally reconstructed surface. Once the reconstruction is lifted, the Au(111) forms islands with an average of 1 atomic step height. When the potential is lowered below that of the PZC, the potential-induced surface reconstruction results in a more disoriented pattern than the thermally reconstructed surface. ECSTM images at different potentials are correlated with the voltammogram to understand the time and potential dependence of the surface. This correlation has led to the development of a potential window technique that can be used to reveal the surface structure of Au(111) based on observing the changes in PZC in the voltammogram. This method provides an indirect approach to understanding the surface structure without always relying on ECSTM. From the voltammogram, we also observed that anions (SO_4_^2−^, CH_3_SO_3_^−^, ClO_4_^−^, F^−^) interact more strongly with the Au(111) surface than the alkali cations. The cation capacitance peak shape does not depend strongly on the identity of the alkali metal cation (Li^+^, Na^+^, K^+^). However, the anion capacitance peak depends strongly on the anion identity. It suggests that some level of specific adsorption cannot be excluded, even for anions that are traditionally not considered to adsorb specifically (perchlorate, fluoride).

## Abstract 2

Electric double-layer capacitance plots on Au(111) are studied to better understand the role of water, cations, and anions, coupled with *in situ* scanning tunnelling microscopy under electrochemical conditions (ECSTM) to track the mobile surface.

## Graphical abstract

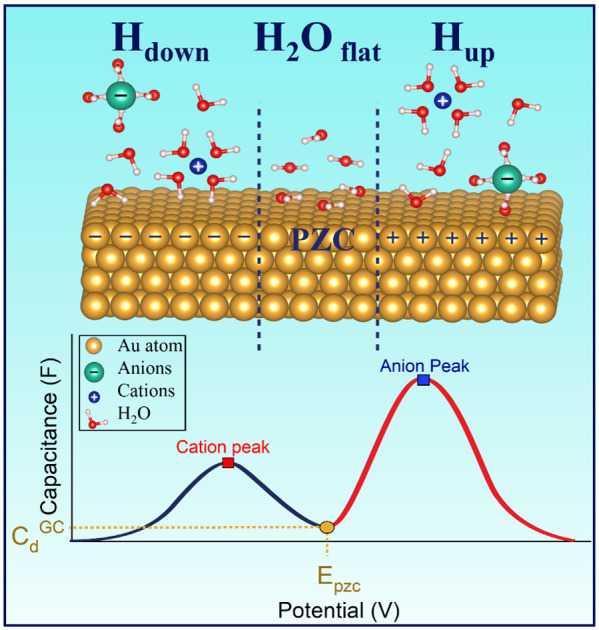

## Introduction

In an electrochemical system, charge-transfer reactions occur at the electrode–electrolyte interface. Therefore, the atomic-scale structure of this interface should substantially impact the rate of electrode reactions. As a result, there is a renewed interest in studying the electric double-layer to optimise reaction conditions.^1–5^ The basic theory of the EDL was built on studies of the mercury–electrolyte interface.^6^ Later, these studies were extended to single-crystal sp-metal surfaces, such as silver and gold, to study the aqueous electrolyte–solid surface interface.^7^ Early on, it became evident that not only the metal identity but also the atomic-scale surface structure influences the double-layer capacity and the so-called potential of zero charge (PZC).^8–12^ At the PZC, the net free charge on the electrode surface is zero, meaning the electrode has no excess positive or negative charge. As a result, the PZC plays an important parameter in defining the electrostatic interactions at the interface. In this regard, single-crystal gold surfaces are the most studied solid electrodes for understanding the structure of their well-defined crystal faces, not only for their broad double-layer region, but also for their relative ease of handling and preparation.^11–15^ Furthermore, gold is a suitable electrocatalyst for fundamental studies of the electrochemical CO_2_ reduction (CO2RR).^16^ It is well known in the literature that hydrogen evolution reduction (HER) is a competing reaction and greatly lowers the faradaic efficiency of CO2RR.^16,17^ Recently, the importance of metal cations present in the electrolyte for both HER and CO2RR was emphasised.^17–19^

Since these metal cations reside in the EDL, the study of the EDL of single crystal gold electrodes is an important topic in fundamental surface electrochemistry. Regarding the low-index facets Au(110), Au(100) and Au(111), a consensus has been reached that their surfaces reconstruct; even Au(111), the most closely packed surface, is known to reconstruct.^20,21^ However, the surface reconstruction of Au(111) has less pronounced features in the voltammetry than the reconstruction of Au(100)^22–26^ Hamelin *et al.* hypothesised that the hysteresis observed in the positive and negative-going scan in double-layer measurements of Au(111)^8,14,27^ could be due to potential-induced changes in the surface structure, as had been reported for Au(100).^22–26^ Kolb *et al.* demonstrated the presence of the potential-induced surface reconstruction in Au(111) by performing *ex situ* reflection high-energy electron diffraction studies (RHEED). They linked changes in the potential of zero charge (PZC) in the positive and negative-going scans of a voltammogram to the lifting of the reconstruction of Au(111).^21^

To understand the lifting of the reconstruction and whether or not the potential-induced surface reconstruction exists, and the corresponding kinetics involved, further studies have been done by *in situ* scanning tunnelling microscopy (STM),^24,28–34^ atomic force microscopy (AFM),^31,35^ density functional theory (DFT) calculations,^33^*in situ* surface X-ray scattering^25,36,37^ and by employing structure-sensitive probe reactions.^20,21,24,28,38^ For Au(100), it is known that the reconstruction lifts due to anion adsorption after applying a sufficiently positive electrode potential. The reconstruction can reappear by applying an electrode potential lower than that of the PZC.^28,39–41^

For Au(111), the presence of the herringbone reconstruction, the lifting of this reconstruction and the occurrence of a potential-induced reconstruction have been reported previously. However, most of the work related to surface reconstruction has been done in H_2_SO_4_, *i.e.* with specifically adsorbing sulfate anions.^28,29,38^ There are relatively fewer studies of the Au(111) electrode in perchloric acid, especially concerning surface reconstruction phenomena. Au(111) in perchloric acid is the model electrode–electrolyte interface for double-layer studies and catalytic reactions like HER.^18,38,42^ It is still unclear to what extent perchloric acid adsorbs on gold and whether this affects the kinetics of lifting of the surface reconstruction.^8,13,27,43,44^ Many DFT calculations of adsorption on gold single crystals are based on an ideal (111) surface,^45^ which may not be the case at every potential, as the Au(111) has the tendency to reconstruct, and the atoms on a Au(111) surface are very mobile. Therefore, it is important to know exactly what the structure of the surface is at a given potential, whether or not it changes when in contact with different electrolytes, and how it affects the electrochemical fingerprint in terms of the blank cyclic voltammetry as well as the reactivity for probe reactions.

We show here that the Au(111) electrode in dilute perchloric acid shows the expected “camel shape” of the potential-dependent double-layer capacity,^5,35,46–48^ with the negative potential peak sensitive to cations and the positive potential peak sensitive to anions.^4^ However, the exact double-layer capacity and double capacity curve depend on the surface reconstruction, with a small negative shift in PZC for the unreconstructed Au(111). In perchloric acid, the lifting of the thermal reconstruction is faster than the re-emergence of the potential-induced surface reconstruction. No clear transition potential can be identified in either case. Our work provides the basis for future double-layer studies of Au(111) and other Au surfaces at different pH and electrolyte compositions with respect to the types of cations and anions.

## Materials and methods

### Chemicals

The electrolytes were prepared from high-purity reagents HClO_4_ (60% Suprapur, Sigma-Aldrich), NaClO_4_ (99.99%, Sigma-Aldrich), LiClO_4_ (99.995%, Sigma-Aldrich), CsClO_4_ (99.99%, Sigma-Aldrich), H_2_SO_4_ (98%, EMSURE, Merck), CH_3_SO_3_H (99%, Sigma-Aldrich), HF (40%, Suprapur, Sigma-Aldrich) and Ultrapure water (Milli-Q gradient, ≥18.2 MΩ cm, TOC < 5 ppb). Argon (Ar) gas (6.0 purity, Linde) purges the electrolytes. We note that CsClO_4_ generally has a high content of impurities. Therefore, we purified CsClO_4_ by multiple recrystallizations in ultrapure water.

### General electrochemical methods

The glassware was stored overnight in a 1 g L^−1^ solution of KMnO_4_ and H_2_SO_4_. Before use, the glassware was submerged in a mixture of H_2_SO_4_ and H_2_O_2_ to remove any trace of KMnO_4_ and MnO_2_. Then, the glassware was washed and boiled six times using ultrapure water. All the electrochemical measurements were carried out using a Biologic(VSP-300) potentiostat. The measurements were carried out in a homemade borosilicate glass cell in which the reference electrode was separated from the working compartment with the help of a Luggin capillary. A shunt capacitor of 10 μF was connected with the RHE, and a Au wire, 99.99% purity, was used as a counter electrode. Before every experiment, the electrode was purged with Argon (Ar) gas for approximately 20 min. A constant Ar gas blanket above the electrolyte was also maintained throughout the measurements to prevent O_2_ from entering the cell.

A single crystal with a 2 mm thickness and 5 mm diameter (purity 99.9%, Mateck GmbH) was used. It was annealed and then cooled down before adding a droplet of degassed Milli-Q water. Annealing without quenching with water resulted in a very high and distorted capacitance curve in the first cycle of cyclic voltammetry, indicating the presence of impurities from air or the heating flame. It is crucial to emphasise that the Au(111) single crystal should be annealed to a point where it slightly glows, but the red-hot flame should be cooled down before making it come into contact with the water.

### Capacitance plots

The Au(111) single crystal was brought into the hanging meniscus configuration under a controlled potential of 0.06 V *vs.* RHE. Some of the measurements described below were done in a very low electrolyte concentration with no additional supporting electrolyte. The distance between the working electrode and the tip of the Luggin capillary was kept approximately 1 cm apart. The Ohmic drop of the electrolyte was determined by carrying out electrochemical impedance spectroscopy (EIS) at 0.06 V (*vs*. RHE; double-layer region), and 85% was corrected during CV measurement and for 100% after the measurement. It is important to note that the current was extremely low for some measurements despite a high solution resistance; correcting it did not give a major shift in the potential when the 85% ohmic drop correction was not applied. The current recorded from the voltammograms was converted to capacitance and normalised by the surface area of the single crystal.
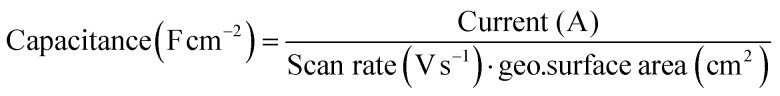
with capacitance in Farads per square centimeter (F cm^−2^), current in amperes (A), scan rate in volts per second (V s^−1^), and geo surface area is the geometric surface area of the electrode (in cm^2^).

### EC-STM

*In situ* scanning tunnelling microscopy performed under electrochemical conditions (EC-STM) has been performed using a home-built instrument.^49^ The design is similar to the setup suggested by Drake *et al.*,^50^ but with some modifications.^50^ The sample is placed in a face-upward configuration, and a flow cell is installed at the top. A hole at the centre of the flow cell helps electrolyte convection and facilitates the tip's approach to the sample surface. The system is sealed with an O-ring to avoid leakage during long experiments. The distance between the working and reference electrodes is about 7 millimetres to minimise the ohmic drop during the voltage sweep, and the cell's shape is designed to allow for a low residence time. Using the pulling–cutting method, the tips were produced from a platinum/iridium wire. Then, a hot melt adhesive layer was added to the tip to reduce the faradaic current. A gold wire was used as the counter electrode, and a reversible hydrogen electrode was used as a reference electrode. A home-built potentiostat changes the tunnelling bias without affecting the electrochemical voltage and *vice versa*. The images were recorded in a constant current mode in the 100–150 pA range. The tunnelling bias was in the range of −10 to −30 mV. The applied current setpoint was zero to retract the tip during the voltage sweep. Then, the tunnelling current on the tip appeared by increasing the setpoint.

## Results and discussion

**Fig. 1:**
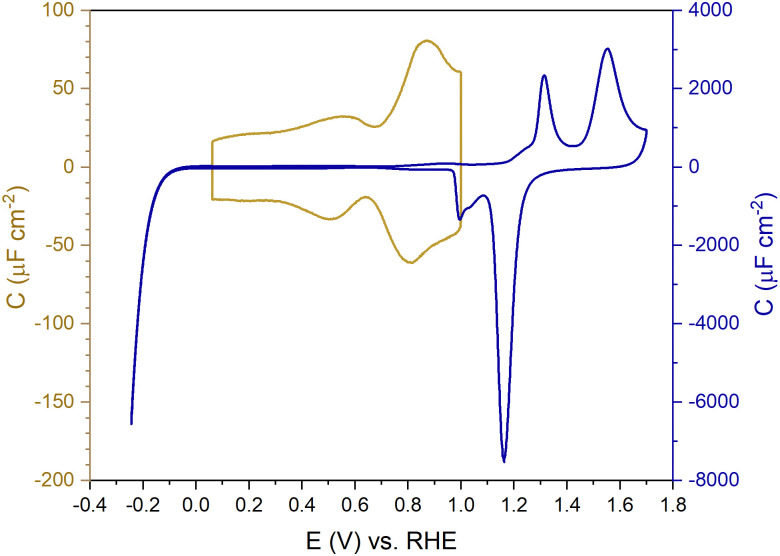
Capacitance *vs.* potential curve showing the double-layer region (golden) and oxidation and reduction at positive potentials and HER at negative potential (blue) of Au(111) measured in Ar gas-saturated 1 mM HClO_4_ solution—scan rate: 20 mV s^−1^.

Fig. 1 shows the electric-double-layer region (EDL) of the Au(111) electrode, which is shown for a dilute electrolyte concentration of 1 mM HClO_4_. The EDL window of Au(111) is between 0 V and 1.0 V *vs.* RHE; above this potential, the pre-oxidation starts, increasing the potential further results in surface oxidation. The oxidation–reduction peaks for oxide formation and oxide reduction correspond well to the published CVs for Au(111) in perchloric acid.^14,27^ The low electrolyte concentration was chosen to present a clear minimum in the differential capacitance, corresponding to the potential of zero charge (PZC). This PZC also corresponds well with the literature value.^5^

Cyclic voltammogram features are very sensitive to the Au(111) single-crystal preparation method when measuring the EDL. The surface of the Au(111) single crystal can be reconstructed under the influence of temperature, or the reconstruction can be induced by applying an electrode potential negative of the PZC. It has been well reported in the literature that the thermally reconstructed structure is the more stable configuration with a 
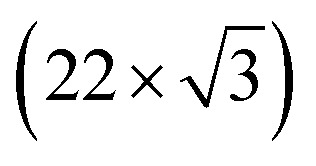
 reconstruction with compression in [110] direction, which leads to the expulsion of adatoms, leading to islands covering *ca.* 4% of the surface upon lifting of the reconstruction.^21,51^

**Fig. 2:**
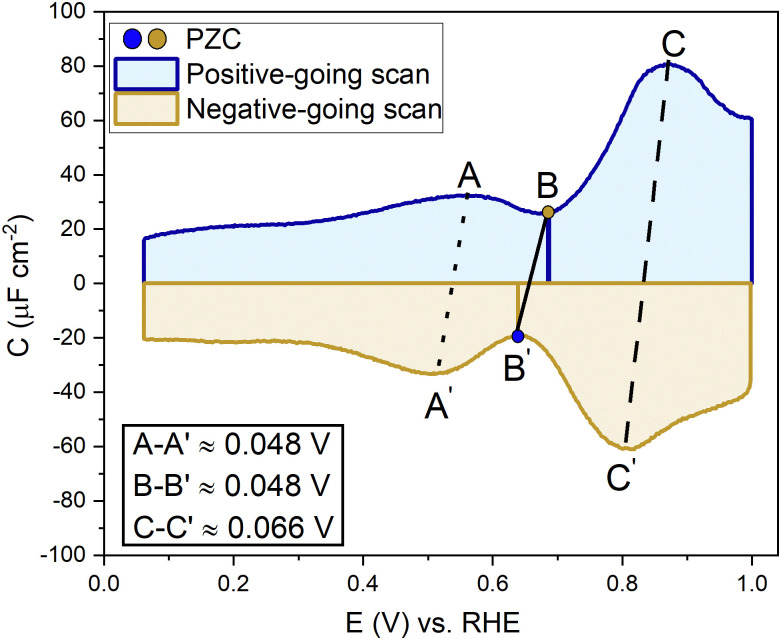
Differentiating between the forward scan (positive potential sweep) and backward scan (negative potential sweep) of the double-layer region, measured in an Ar gas-saturated 1 mM HClO_4_ solution—scan rate: 20 mV s^−1^.

Fig. 2 shows a clear hysteresis between the positive-going and negative-going scans, with a shift in the potential of zero charge (PZC). Hamelin also reported this hysteresis for the Au(111) electrode by observing a shift in the adsorption peak of halide in diluted halide-containing electrolytes (halides are known to adsorb specifically on Au(111) surfaces).^52^ She suggested that this hysteresis was due to the re-arrangement of the surface atoms and that the potential range (be it negative or positive with respect to the PZC) did not affect this hysteresis unless the potential range where the hysteresis started to occur was eliminated.^14,21^ Kolb and co-workers performed experiments with Au(111) in 10 mM HClO_4_ acid. They linked the shift in the PZC to the re-arrangement of atoms, more specifically due to the lifting of reconstruction of the Au(111) surface at potentials positive of the PZC.^21^

Kolb and Schneider showed that a thermally reconstructed Au(111) surface has a more positive PZC than a surface reconstructed under the influence of negative potential after lifting the thermal reconstruction.^21^ It is important to note that the lifting of the reconstruction is a scan rate-dependent process. Depending on the scan rate and the positive electrode potential limit chosen, the lifting of the thermal reconstruction can vary. Also, if the single crystal has defects, that can influence the number of voltammetric cycles required before the reconstruction is lifted all over the surface. Fig. S1 (ESI†) shows EC-STM images of a case where the reconstruction of the Au(111) did not get completely lifted homogeneously throughout the whole surface, leaving patches of reconstruction visible, which in the voltammograms in Fig. S2 (ESI†) can be seen by a small peak appearing before the PZC, slowly diminishing in subsequent other cycles. It shows the presence of more than one surface structure with presumably different PZCs.

**Fig. 3:**
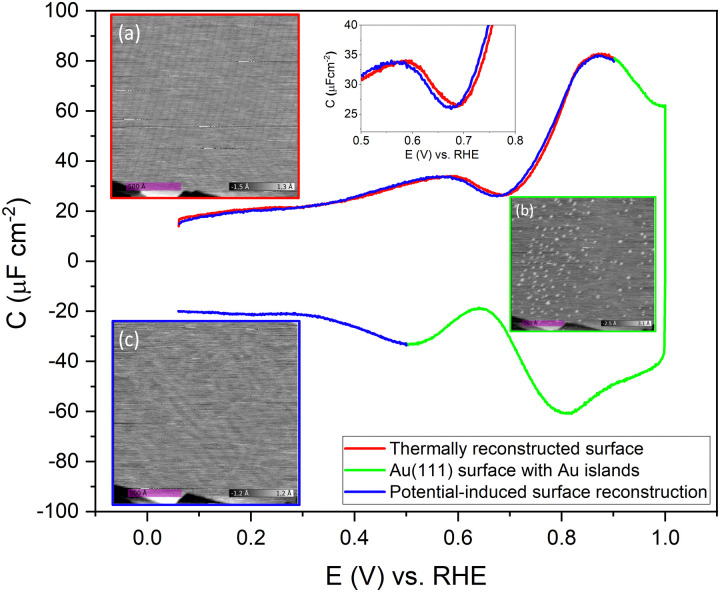
Evolution of the Au(111) electrode surface during CV after initial thermal annealing, as imaged by EC-STM. The images (a) at a potential of 0.06 V *vs.* RHE at the beginning of the positive-going scan, showing the reconstruction lines, (b) at a potential of 1.0 V *vs.* RHE after the positive-going scan, showing a Au(111) surface with small islands, and (c) at a potential of 0.06 V *vs.* RHE after the negative-going scan showing the potential-induced reconstruction, measured in an Ar gas-saturated 1 mM HClO_4_ solution, 20 mV s^−1^.

To confirm the nature of the hysteresis in the cyclic voltammetry, we have performed EC-STM experiments on Au(111) in 1 mM perchloric acid. Fig. 3 summarises the main results. The EDL of Au(111) can be divided into three regions. Starting with a freshly thermally annealed Au(111) crystal (Fig. 3a), the surface of Au(111) is reconstructed, as identified by double rows, that can form a herringbone structure.^33,38,53^ Once this reconstruction is lifted, at a potential positive of the PZC, the surface is an unreconstructed Au(111) covered with small Au islands caused by Au atoms expelled from the surface by the lifting of the reconstruction (Fig. 3b). The reconstruction reappears by applying or scanning the electrode potential negative of the PZC. The resulting potential-induced surface reconstruction (Fig. 3c) is more disordered compared to a thermal reconstruction (Fig. 3a), and the lines also appear broader than for a thermally reconstructed surface. On the basis of these results, we conclude that the capacitance minimum in the positive-going scan corresponds to the PZC of the reconstructed surface, whereas the capacitance minimum in the negative-going scan corresponds to the PZC of the unreconstructed Au(111) surface. Hence, the PZC of the unreconstructed Au(111) surface is slightly less positive than that of the reconstructed Au(111) surface.

**Fig. 4:**
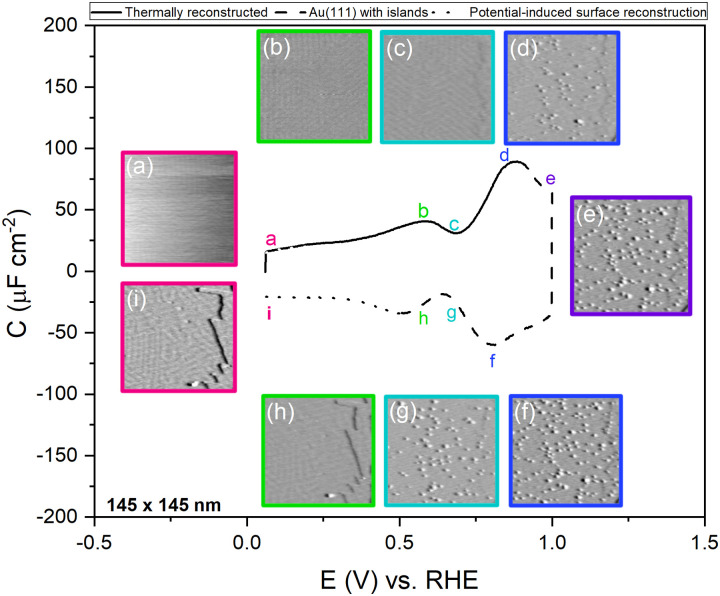
EC-STM images of Au(111) in 1 mM perchloric acid solution at different potentials of the CV; the CV is measured in an Ar gas-saturated solution—scan rate: 20 mV s^−1^. Positive-going scan: (a) thermally reconstructed surface at 0.06 V, (b) 0.6 V, (c) 0.7 V, (d) Au(111) with islands at 0.9 V, (e) 1.0 V. Negative-going scan: (f) 0.9 V, (g) 0.7 V, (h) potential-induced surface reconstruction at 0.6 V and (i) 0.06 V. All potentials *vs*. RHE.

To study the time dependence of the formation of these three different structures in more detail, we have performed EC-STM experiments on Au(111) in 1 mM perchloric acid at different potentials, as shown in Fig. 4. The freshly flame-annealed Au(111) is contacted with the electrolyte at 0.06 V. The surface exhibits the well-known herringbone reconstruction (Fig. 3a and 4a). The structure remains the same when the potential is increased to 0.6 V (Fig. 4b) and then to 0.7 V (Fig. 4c). If the potential is further increased in steps of 0.1 V from 0.7 (PZC) to 0.9 V, the herringbone structure disappears, and the reconstruction starts lifting. The new surface is that of a Au(111) surface with Au islands (Fig. 4d). The reconstruction gets completely lifted once the potential increases to 1.0 V (Fig. 4e). As the potential is lowered in the negative-going scan, the reconstruction takes place, and adatoms from the islands become incorporated again in the surface. This process is inherently slow and yields a reconstructed surface which looks different from a thermally annealed surface. Once the potential is decreased below the PZC, the last remaining Au islands are seen at a potential of *ca.* 0.65 V. After that, the reconstruction starts to appear, and the newly reconstructed surface can be seen in Fig. 4h at a potential of 0.6 V.

This potential-induced reconstruction (Fig. 3c and 4i, both measured at a potential of 0.06 V) appears more disordered with a non-uniform structure. The formation of this reconstructed surface appears to be kinetically slower than the lifting of the reconstruction, as the reconstruction lifts between 0.7 V and 1 V, whereas a larger potential window is necessary for the electrochemical reconstruction to appear, *i.e.* between 0.6 to 0.06 V, where the potential was held for the same duration of time at each potential. The surface was also subjected to negative potentials up to −0.1 V (Fig. S3 left, ESI†). The potential was then increased again from –0.1 V to 1 V to lift the electrochemical reconstruction (Fig. S3, right-hand side, ESI†), and then the potential was lowered back to 0.06 V. The potential-induced reconstruction features seem more pronounced under these conditions, but further experiments will be needed to address this in more detail.

**Fig. 5:**
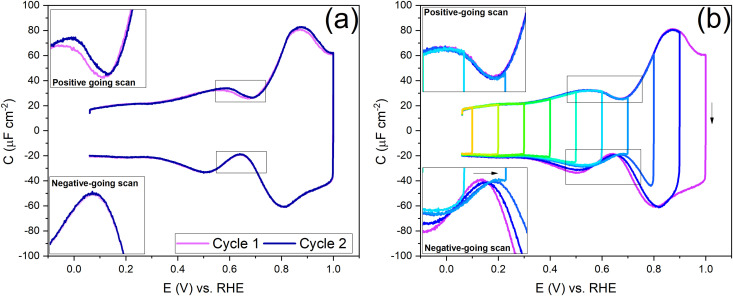
(a) The first two cycles between 0.06 and 1 V indicate the difference in the capacitance and PZC of subsequent positive-going and negative-going scans. (b) Effect of the potential window on cycling in the double-layer region. Purple is the first scan; orange is the last scan—measured in an Ar gas-saturated 1 mM HClO_4_ solution, 20 mV s^−1^.

To understand the role of the potential window on the surface reconstruction, we have measured the double-layer capacitance curves in so-called “window opening” experiments. Fig. 5a shows the first two cycles of the windowing experiment, starting with a freshly thermally reconstructed Au(111) single crystal. The first cycle has a capacitance minimum at 0.69 V *vs.* RHE (0.51 *vs.* SHE), and in cycle 2, the capacitance minimum in the positive-going scan has shifted to a lower value of 0.674 V *vs.* RHE (0.497 *vs.* SHE). It is important to note that in the negative-going scan, the capacitance minimum is around 0.64 V. *vs.* RHE (0.46 *vs.* SHE); it does not change within the first two cycles.

Fig. 5b shows the changes in the double-layer capacitance and PZC within potential windows with a successively less positive vertex potential. These results show that the PZCs of the potential-induced surface reconstruction remain the same. When one reduces the positive limit of the potential in successive scans, barely any change is observed in the positive-going scan, but for the negative-going scan, as the positive limit of the cycle is lowered, the lifting of the reconstruction is prohibited, and the PZC shifts to more positive potentials, as can be seen in the negative-going scan. Therefore, we conclude that the PZC of the potential-induced surface reconstruction of Au(111) is 0.674 V *vs.* RHE (0.497 V *vs.* SHE), whereas the PZC of a Au(111) (with islands resulting from the lifting of the reconstruction) is 0.64 V *vs.* RHE (0.46 V *vs.* SHE). When cycling in the region negative of the PZC (Fig. 5b and Fig. S4, ESI†), where the electrode potential stabilizes the reconstructed surface, the scans are symmetric and, hence, reversible.

**Fig. 6:**
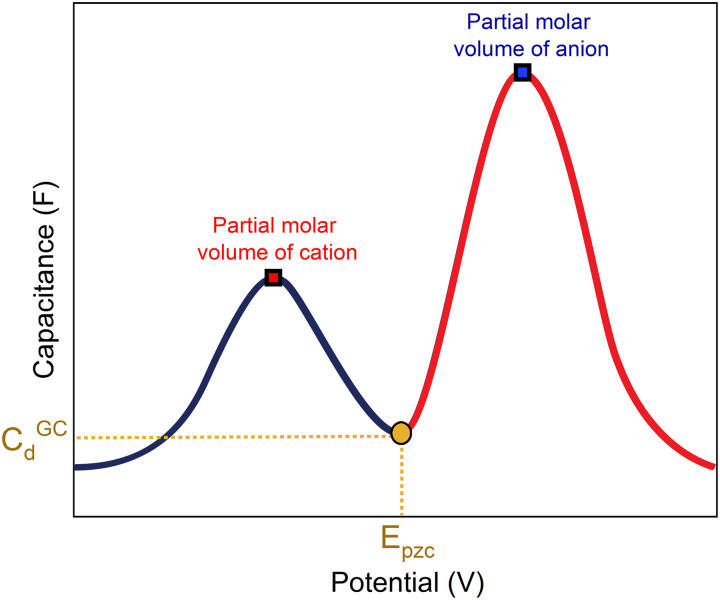
Generalized picture showing a camel-shaped capacitance plot of the double-layer region.

A capacitance curve of the EDL region in a low electrolyte concentration generally shows a so-called “camel plot”, as illustrated schematically in Fig. 6.^4,46^ At low electrolyte concentrations, one expects a minimum in the capacitance at the PZC, which should correspond to the differential capacitance predicted by the Gouy–Chapman theory (*C*_d_^GC^).^54^ Positive and negative of the PZC, there are two peaks corresponding to anions and cations populating the electric double layer, respectively. The intensity of the peaks has been suggested to scale inversely with the size or partial molar volume of the (solvated) ion: the smaller the (solvated) ion, the more ions fit into the double layer, and hence the higher the differential capacitance (peak).^4^ The potential difference between the peaks depends on the concentration of the anions and cations, as well as on the strength of the interaction of the solvent with the surface: the peaks shift closer together (the cation peak shifting positively, the anion peak shifting negatively) at a higher electrolyte concentration, and the peaks shift further apart when the solvent molecules have a stronger interaction with the interface.^4^ Alternatively, the capacitance peaks have been attributed to a decrease in the local dielectric constant as a result of the high electric field; specifically, Shin *et al.* have ascribed the capacitance peak negative of the PZC to this dielectric saturation effect.^55^ At a sufficiently high electrolyte concentration, the peaks will overlap, and the capacitance curve typically takes a bell-shaped form.^46^

**Fig. 7:**
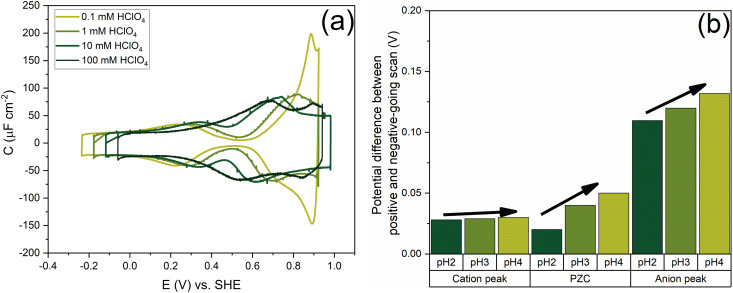
Comparison of increasing HClO_4_ concentration from 0.1 mM (pH 4) to 100 mM (pH 1)—(a) the voltammogram at 20 mV s^−1^ (b) the difference in the peak potential of the cation and anion-related peaks in the positive and negative-going scan and the difference in the PZC, at pH 2, pH 3 and pH 4.

**Fig. 8:**
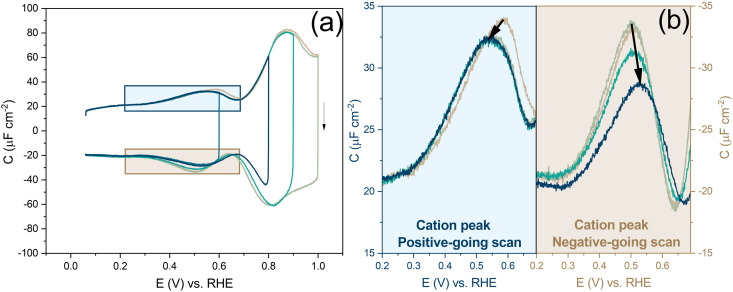
(a) Cycling and lowering the positive vertex potential to hinder the lifting of the reconstruction. (b) Zoomed-in cation peak in the positive and negative going scan, showing the effect of lowering the positive vertex potential, measured in an Ar gas-saturated 1 mM HClO_4_ solution, 20 mV s^−1^.

**Fig. 9:**
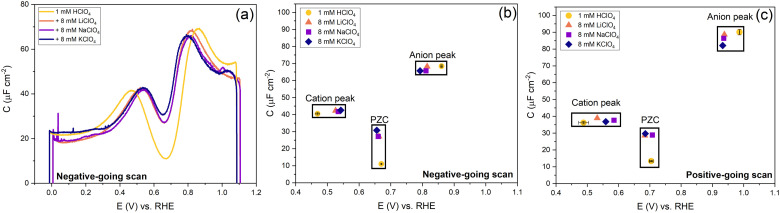
Effect of cations and anions on the peak, PZC capacitance and potential. (a) The potential-dependent capacitance of Au(111) from the negative-going scan is shown. (b) Cation peak capacitance, anion peak capacitance and PZC from negative-going scan and (c) positive-going scan. The full cyclic voltammogram is shown in Fig. S5 (ESI†). All curves were measured in 1 mM HClO_4_ background electrolytes in an Ar gas-saturated solution, and the scan rate was 20 mV s^−1^.

**Fig. 10:**
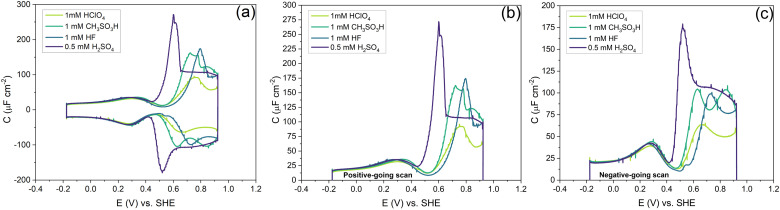
(a) The effect of changing the anion. (b) The zoomed-in version of the positive-going scan shows thermally reconstructed Au(111) features. (c) The zoomed-in version of the negative-going scan showing Au(111) features. Measured in an Ar gas-saturated solution—scan rate: 10 mV s^−1^.

Fig. 7 shows the capacitance curves in a concentration range from 0.1 mM (pH 4) to 100 mM (pH 1) HClO_4_. It confirms the transition from a camel-shaped capacitance curve to a bell-shaped capacitance curve with increasing electrolyte concentration.^46,48^ A hysteresis is observed when comparing the positive and negative-going scans due to the surface reconstruction, as already shown in Fig. 2. If the hysteresis was only due to the difference in the work function between the different surfaces, the variation in the PZC and the anion- and cation-related peak potentials between the positive and negative-going scans should be the same. However, as shown in Fig. 8b, a clear variation is seen in the potential differences of the PZC compared to the anion peak potentials in the positive and negative-going scan in different concentrations of HClO_4_. The hysteresis in PZC and anion peak increases as the HClO_4_ concentration decreases. This could be due to the reconstructed and unreconstructed surface interacting differently with interfacial water or with the anion. The cation potential peaks have the smallest difference as the surface is supposed to be reconstructed in the corresponding potential region, in both scan directions. The anion peak at pH 4 clearly stands out from the rest while scanning in both directions. This change could be due to there not being enough perchlorate anions to facilitate the lifting of the reconstruction. The reconstruction gets lifted once the potential increases sufficiently positive, causing an increase in the surface charge. Whether or not perchlorate is or needs to be specifically absorbed to facilitate the lifting of the reconstruction is an issue that we will return to in relation to Fig. 9 and 10. One other aspect can be the onset of adsorbed hydroxyl species, but future experiments in neutral and alkaline media will be needed to elucidate this.

Before looking into the Au(111) data with electrolytes containing different anions and cations, it is important to compare the generalized model with a voltammogram of Au(111) in 1 mM perchloric acid and highlight the impact the potential dependent surface structure can have on the cation and anion peaks. Fig. 8a shows that the surface structure influences the potential and capacitance of the anion and cation peaks. Focusing on the region positive of the PZC, the anion peak capacitance in the first two consecutive cycles is the same for both positive and negative-going scans, but the capacitance is significantly lower in the negative-going scan in comparison to the positive-going scan (*ca.* 61 μF cm^−2^*vs. ca.* 80 μF cm^−2^ peak capacitance). The reason for this discrepancy has likely to do with effect of anions on the (rate of) lifting of the reconstruction. When the lifting of the reconstruction is hindered by lowering the positive vertex potential to 0.8 V, the cation-related peak potential becomes reversible (see Fig. 8b), indicating that the surface structure is the same in both the positive and negative-going scans. However, we note that there is still a small discrepancy in peak capacitance between positive- and negative going scan (*ca.* 32 μF cm^−2^*vs. ca.* 28 μF cm^−2^), the reason for which remains unresolved.

Compared to the intensity of the anion peak, the capacitance of the cation peak is almost half that of the anion peak, suggesting that the effective molar volume of the cations is quite a bit higher than that of the anions. Fig. 8b highlights the cation peaks in the positive and negative-going scans. In the positive-going scan, when the thermally reconstructed surface (cycle 1) is compared to the potential-induced surface reconstruction (cycle 2), the cation peak is shifted to lower potential on the latter. This shift agrees with the shift in PZC shown in Fig. 5a. Note that the anion peak potential shows no shift in the positive-going scan, because the surface reconstruction is lifted in both cycles. Also, the cation (or proton) peak capacitance is somewhat lower for the potential-induced surface reconstruction for reasons that we have not elucidated. In the negative-going scan, the cation peak potential follows the shift in the PZC, the potential of which increases with less positive vertex potential.

In Fig. 9, we illustrate the effect of different cations on the capacitance against a background electrolyte of 1 mM HClO_4_. First of all, on the addition of the cations, the shift of the PZC is negligible (at best, it seems to shift very slightly negatively). The capacitance at PZC increases from *ca.* 10 to *ca.* 25–30 μF cm^−2^ by the addition of 8 mM alkali perchlorate. According to the Gouy–Chapman theory, the capacitance at 1 mM and 9 mM should be 7.2 μF cm^−2^ and 21.6 μF cm^−2^, respectively. Both the cation peak and the anion peak shift towards the PZC when 8 mM XClO_4_ (where X: Li, Na, K) is introduced into a solution with 1 mM HClO_4_, in agreement with the expectation of how the peaks in a camel capacitance plot should change with an increase in electrolyte concentration. Surprisingly, the capacitance of the cation-related peak remains essentially the same for all alkali cations, despite their different hydration radii. This could mean that changes in the interfacial dielectric constant could be a more likely explanation of the cation-related capacitance peak.^55^ The intensity of anion peak should remain the same with the addition of XClO_4_ electrolyte. It appears to very slightly decrease in both the positive going scan and negative going scan compared to the cycle with only 1 mM HClO_4_ background electrolyte present. This is in agreement with Fig. 7 for the anion peak in different pH of HClO_4_ acid, with the exception of pH 4. The difference with pH 4 is striking and reproducible, as well as somewhat puzzling. Since higher capacitance peaks could signify the effect of specific adsorption (see *e.g.* the curves for sulfate-containing electrolyte in Fig. 10), it seems that we cannot entirely rule out some level of perchlorate specific adsorption at higher potential. However, whether perchlorate (or anions in general) facilitate the lifting of the reconstruction by stabilizing the surface charge through electrostatic interactions or by some level of specific adsorption at potentials positive of the PZC, is also not fully clear.

Fig. 10 shows the effect of different anions on the capacitance curve in a 1 mM solution with protons as the cation. Fig. 10a shows that the cation (*i.e.* proton) peak potential and capacitance barely change with the identity of the anion. The anion peak potential and intensity clearly depend on the anion, with the highest capacitance and lowest peak potential for SO_4_^2−^. The strong effect of sulfate is due to the specific adsorption of sulfate on Au(111), which is well-known.^56^ The capacitance minimum shifts to lower potential in the order of SO_4_^2−^ < CH_3_SO_3_^−^< ClO_4_^−^ < F^−^, with the latter three anions showing only minimal shifts, suggesting their lack of strong specific adsorption, at least around the PZC. For methane–sulfonate, we expect that there could still be some weak specific adsorption.^57^ If we assume fluoride and perchlorate ions do not adsorb specifically, the higher differential capacitance for HF would suggest that (hydrated) fluoride has a smaller partial molar volume than (hydrated) perchlorate.

Comparing the anion capacitance peaks from Fig. 10b and c (*i.e.*, obtained from the positive-going scan and the negative-going scan, respectively), it is evident that capacitance in the positive-going scan is always substantially higher than in the negative-going scan. From the EC-STM data we know that in the positive-going scan, the lifting of the reconstruction starts to takes place. As mentioned previously, it is generally assumed that this lifting is promoted by the specific adsorption of anions.^58^ We cannot exclude the specific adsorption of anions on low-coordination sites generated (or even needed) for the lifting of the reconstruction. Combined with the observation that there is a very strong discrepancy in peak capacitance for perchlorate at pH 4 (see Fig. 7), the idea that perchlorates or fluorides might specifically adsorb at higher potentials, perhaps at certain sites, can not be dismissed. Further research would be needed to study by which specific mechanism anions would facilitate the lifting of the reconstruction.

## Conclusions

In this paper, we have studied the electric double-layer of a Au(111) surface in weakly and non-adsorbing electrolytes by capacitance and EC-STM measurements. The Au(111) single-crystal surface structure is highly sensitive to the preparation method and the electrode potential applied. The surface structure right after thermal annealing has the well-known herringbone reconstruction pattern. The reconstruction is lifted in the positive-going scan at potentials positive of the potential of zero charge (PZC). Since the lifting of the reconstruction also occurs in perchlorate electrolyte, it seems to be mainly driven by the positive surface charge, although we cannot exclude the effect of a small extent of specific adsorption on certain sites. The surface reconstructs again in the negative going-scan. The slow kinetics of the lifting and subsequent reconstruction leads to the observed irreversibility in the capacitance curves: the PZC of the reconstructed surface is 0.51–0.497 V *vs.* SHE (depending on whether the surface has been reconstructed thermally or potential-induced, resp.), whereas that of the unreconstructed Au(111) surface with Au islands is 0.46 V *vs.* SHE. The capacitance curve of Au(111) in a low-concentration electrolyte follows the “camel-shaped” model of the double layer. The cation capacitance peak shape and intensity are insensitive to the identity of the alkali metal cation, suggesting that this peak is due to a change in interfacial dielectric constant. On the other hand, there is a strong dependence of the peak shape and capacitance on the anion type for the anion capacitance peak. For sulfate and methane–sulfonate, this may be (partially) explained by specific adsorption. For fluoride and perchlorate, for which we do not expect strong specific adsorption, the peak intensity should correspond to the different (hydrated) anion sizes. Still, there is substantial irreversibility in the anion capacitance peak intensity between positive- and negative-going scans, presumably related to the slowness of the reconstruction of the surface. Therefore, we cannot exclude some level of specific adsorption, even for fluoride and perchlorate.

## Author contributions

Ariba Adnan: writing – original draft, writing – review & editing, methodology, investigation, formal analysis, data curation, visualization, conceptualization. Saeid Behjati: writing – review & editing, data curation, methodology, investigation, formal analysis. Núria Félez-Guerrero: investigation, data curation, validation. Kasinath Ojha: investigation, data curation. Marc T. M. Koper: writing – review & editing, supervision, funding acquisition, formal analysis, conceptualization.

## Data availability

All data will be made available upon reasonable request.

## Conflicts of interest

The authors declare that they have no known competing financial interests or personal relationships that could have appeared to influence the work reported in this paper.

## Supplementary Material

CP-026-D4CP02133A-s001

## References

[cit1] Ojha K., Doblhoff-Dier K., Koper M. T. M. (2022). Proc. Natl. Acad. Sci. U. S. A..

[cit2] Dourado A. H. B. (2022). Electrochem.

[cit3] Schmickler W. (2021). ChemElectroChem.

[cit4] Shatla A. S., Landstorfer M., Baltruschat H. (2021). ChemElectroChem.

[cit5] Ojha K., Arulmozhi N., Aranzales D., Koper M. T. M. (2020). Angew. Chem., Int. Ed..

[cit6] Proskurnin M., Frumkin A. (1934). Trans. Faraday Soc..

[cit7] Hamelin A., Vitanov T., Sevastyanov E., Popov A. (1983). J. Electroanal. Chem. Interfacial Electrochem..

[cit8] Hamelin A. (1982). J. Electroanal. Chem. Interfacial Electrochem..

[cit9] Hamelin A. (1982). J. Electroanal. Chem. Interfacial Electrochem..

[cit10] Valette G. (1982). J. Electroanal. Chem. Interfacial Electrochem..

[cit11] D’Agostino A. T., Ross P. N. (1985). J. Electroanal. Chem. Interfacial Electrochem..

[cit12] Trasatti S. (1985). Mater. Chem. Phys..

[cit13] Schmid G. M., Hackerman N. (1962). J. Electrochem. Soc..

[cit14] Hamelin A., Borkowska Z., Stafiej J. (1985). J. Electroanal. Chem. Interfacial Electrochem..

[cit15] Hamelin A. (1985). J. Electroanal. Chem. Interfacial Electrochem..

[cit16] Monteiro M. C. O., Dattila F., López N., Koper M. T. M. (2022). J. Am. Chem. Soc..

[cit17] Monteiro M. C. O., Goyal A., Moerland P., Koper M. T. M. (2021). ACS Catal..

[cit18] Goyal A., Koper M. T. M. (2021). Angew. Chem., Int. Ed..

[cit19] Xue S., Garlyyev B., Watzele S., Liang Y., Fichtner J., Pohl M. D., Bandarenka A. S. (2018). ChemElectroChem.

[cit20] Tanishiro Y., Kanamori H., Takayanagi K., Yagi K., Honjo G. (1981). Surf. Sci..

[cit21] Kolb D. M., Schneider J. (1986). Electrochim. Acta.

[cit22] Schneider J., Kolb D. M. (1988). Surf. Sci..

[cit23] D’Agostino A. T., Ross P. N. (1987). Surf. Sci..

[cit24] Gnahm M., Berger C., Arkhipova M., Kunkel H., Pajkossy T., Maas G., Kolb D. M. (2012). Phys. Chem. Chem. Phys..

[cit25] Kondo T., Morita J., Hanaoka K., Takakusagi S., Tamura K., Takahasi M., Mizuki J., Uosaki K. (2007). J. Phys. Chem. C.

[cit26] Kondo T., Zegenhagen J., Takakusagi S., Uosaki K. (2015). Surf. Sci..

[cit27] Hamelin A. (1986). J. Electroanal. Chem. Interfacial Electrochem..

[cit28] Kolb D. M. (2000). Electrochim. Acta.

[cit29] Li Z., Han B., Wan L. J., Wandlowski Th (2005). Langmuir.

[cit30] Vaz-Domínguez C., Aranzábal A., Cuesta A. (2010). J. Phys. Chem. Lett..

[cit31] He Y., Borguet E. (2011). J. Phys. Chem. C.

[cit32] Yoshimoto S., Kim Y.-G., Sato K., Inukai J., Itaya K. (2012). Phys. Chem. Chem. Phys..

[cit33] Hanke F., Björk J. (2013). Phys. Rev. B: Condens. Matter Mater. Phys..

[cit34] Hamill J., Zhour K., Diddens D., Baghernejad M. (2022). Electrochem. Commun..

[cit35] Hayes R., Borisenko N., Tam M. K., Howlett P. C., Endres F., Atkin R. (2011). J. Phys. Chem. C.

[cit36] Wang J., Ocko B. M., Davenport A. J., Isaacs H. S. (1992). Phys. Rev. B: Condens. Matter Mater. Phys..

[cit37] Wandlowski T., Ocko B. M., Magnussen O. M., Wu S., Lipkowski J. (1996). J. Electroanal. Chem..

[cit38] Hermann J. M., Abdelrahman A., Jacob T., Kibler L. A. (2020). Electrochim. Acta.

[cit39] Kolb D. (1996). Prog. Surf. Sci..

[cit40] Friedrich A., Pettinger B., Kolb D. M., Lüpke G., Steinhoff R., Marowsky G. (1989). Chem. Phys. Lett..

[cit41] Zei M. S., Lehmpfuhl G., Kolb D. M. (1989). Surf. Sci..

[cit42] Goyal A., Marcandalli G., Mints V. A., Koper M. T. M. (2020). J. Am. Chem. Soc..

[cit43] Zhumaev U. E., Pobelov I. V., Rudnev A. V., Kuzume A., Wandlowski T. (2014). Electrochem. Commun..

[cit44] Zhumaev U. E., Lai A. S., Pobelov I. V., Kuzume A., Rudnev A. V., Wandlowski Th (2014). Electrochim. Acta.

[cit45] Duan Z., Henkelman G. (2018). Langmuir.

[cit46] Kornyshev A. A. (2007). J. Phys. Chem. B.

[cit47] Fedorov M. V., Georgi N., Kornyshev A. A. (2010). Electrochem. Commun..

[cit48] Huang J. (2023). JACS Au.

[cit49] Behjati S. and Koper M. T. M., unpublished work

[cit50] Drake B., Sonnenfeld R., Schneir J., Hansma P. K. (1987). Surf. Sci..

[cit51] Harten U., Lahee A. M., Toennies J. P., Wöll C. (1985). Phys. Rev. Lett..

[cit52] Lipkowski J., Shi Z., Chen A., Pettinger B., Bilger C. (1998). Electrochim. Acta.

[cit53] Seitsonen A. P. (2016). Surf. Sci..

[cit54] Doblhoff-Dier K., Koper M. T. M. (2023). Curr. Opin. Electrochem..

[cit55] Shin S.-J., Kim D. H., Bae G., Ringe S., Choi H., Lim H.-K., Choi C. H., Kim H. (2022). Nat. Commun..

[cit56] Edens G. J., Gao X., Weaver M. J. (1994). J. Electroanal. Chem..

[cit57] Masens C., Ford M. J., Cortie M. B. (2005). Surf. Sci..

[cit58] Magnussen O. M. (2002). Chem. Rev..

