# Supplementary material for: Tracking the surface structure and the influence of cations and anions on the double-layer region of a Au(111) electrode
Source: Phys Chem Chem Phys. 2024 Aug 1;26(32):21419–28. doi: 10.1039/d4cp02133a (PMC11323936; doi:10.1039/d4cp02133a)
Supplement: CP-026-D4CP02133A-s001 [file CP-026-D4CP02133A-s001.pdf]

Supplementary Information (SI) for Physical Chemistry Chemical Physics.
This journal is © the Owner Societies 2024

Supplementary information to

Tracking the surface structure and the influence of cations and anions on the double-layer region of a Au(111) electrode.

Ariba Adnan[a], Saeid Behjati[a], Núria Félez-Guerrero[a,b], Kasinath Ojha[a], Marc T. M. Koper[a]

[a] *Leiden Institute of Chemistry, Leiden University, Einsteinweg 55, 2333 CC Leiden, the Netherlands*
[b] *Chemistry department, Universitat Autònoma de Barcelona, 08193-Bellaterra, Barcelona, Spain*

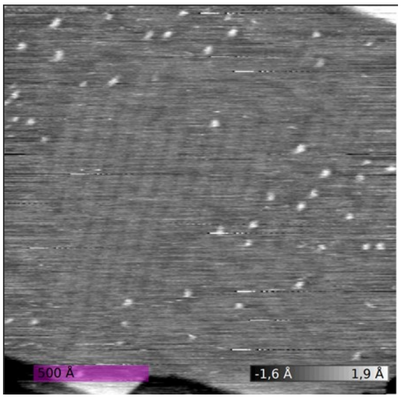

Figure S1: EC-STM images of a thermally annealed Au(111) surface for which the reconstruction did not lift homogeneously upon applying the potential positive of the PZC. The preparation method of the single crystal can influence the surface. If the crystal is not annealed properly, the surface can vary. There may be patches where the surface is still reconstructed and somewhere it is not.

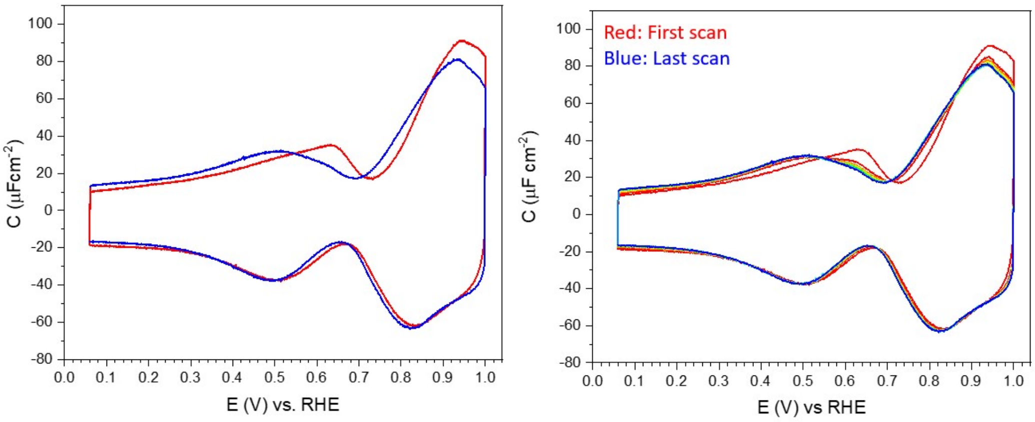

Figure S2: Notably, the reconstruction process is not always homogeneous throughout the crystal. This intriguing observation could be attributed to the annealing process or the potential window chosen, which may have been smaller than optimal. A voltammogram can determine if the crystal is not annealed well. Since the surface is not homogeneous, a peak before the PZC can be seen. This seems to be related to the surface's inhomogeneity as after cycling this peak disappears or after re-annealing.

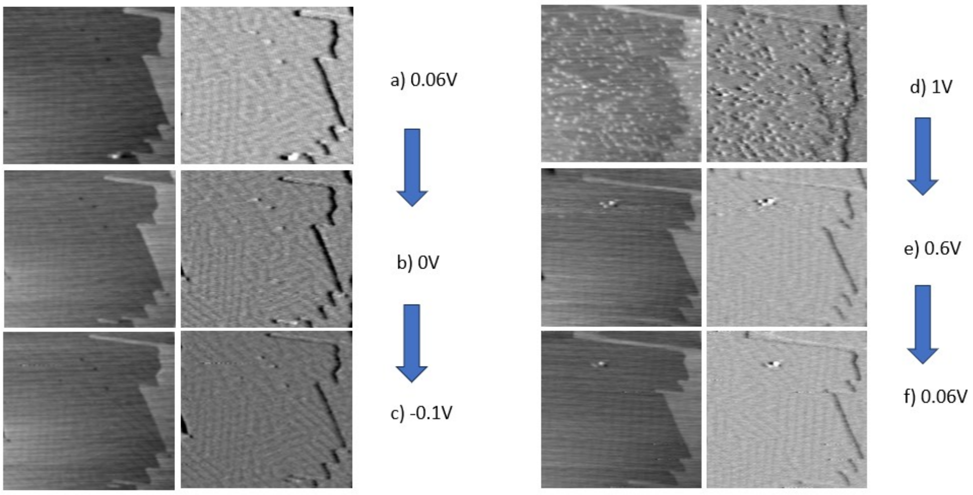

Figure S3: (Left) The reconstruction remains at negative potentials. (Right) The reconstruction is lifted again, and the electrochemical reconstruction is visible. Image size: 145 x 145 nm. The surface is electrochemically reconstructed even at negative potentials (Figure S3c). The pattern appears to be getting slightly more ordered. Upon lifting this reconstruction again (Figure S3d) and inducing reconstruction(Figure S3e), the reconstruction pattern appears to be more ordered.

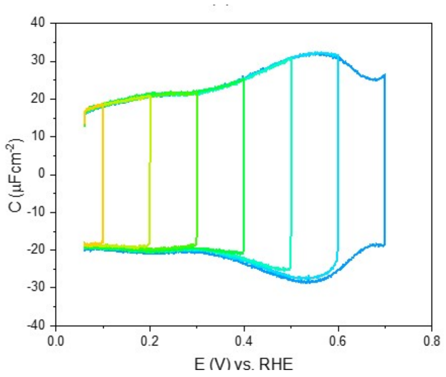

Figure S4: Cycling in the region negative of the PZC, the surface is reconstructed at all potentials, leading to (almost) reversible CVs. —measured in an Ar gas-saturated solution—scan rate: 20 mVs^{-1}. The voltammograms appear symmetrical if the potential window is not increased further than the PZC. This is due to the surface being electrochemically reconstructed both in the positive and negative going scan.

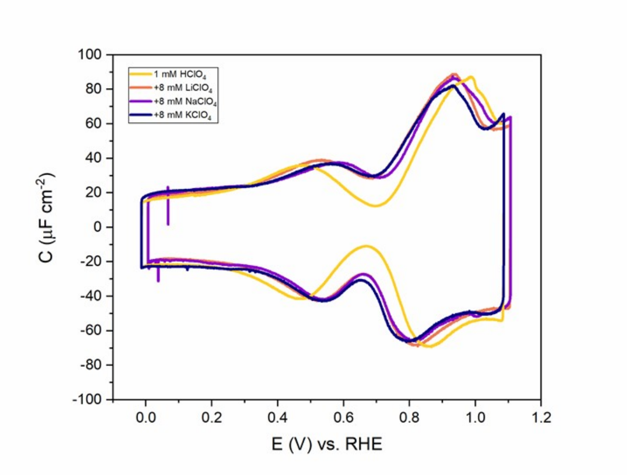

Figure S5: The full cyclic voltammogram showing the effect of cations and anions on the peak, pzc capacitance and potential. The cation peak and the anion peak show a “camel-shaped plot.” The shape of the cation peak does not seem to depend on the alkali cation type.
